# Event and Comment

**Published:** 1909-07-15

**Authors:** 


### DENTAL REGISTER.

Vol. LXIII. July 15, 1909. No. 7.

### EVENT AND COMMENT.

**PROPOSED ENGLISH ANESTHETIC LAW.** In another place of this journal is reprinted an editorial on the proposed law now before the English Parliament intended to confine the administration of nitrous oxide to medically qualified practitioners only. Recently the bill has been amended to include hypodermic injection of cocaine and other toxic analgesics. The fact that in this country nitrous oxid anesthesia is almost exclusively confined to dentists, makes the entire proposition seem ridiculous. Dr. Kirk's editorial makes a strong argument against the enactment of such a law. There can be no question that dentists are more skillful in the technical manipulation of this agent than medical men are likely to become and it is unquestionably a safe and reliable agent for dental analgesia. There can be in our judgment but one good reason given for the enactment of such a law and that is that medical men should be better qualified to diagnose unfavorable or contra indicating infirmities in patients presenting for the anesthesia. But the record of a large number of administrations and the comparatively few accidents with gas is fairly valuable testimony that dentists have either accomplished this satisfactorily, or that there are few infirmities that contraindicate its use. Until it can be demonstrated that the profession of dentistry is manifestly incapable in its use, it would seem that a gross wrong would be done to deprive the afflicted of this great pain relieving agent.

